# Effect of Music-Based Interventions on Dental Anxiety During Restorative Dental Treatment: A Randomized Controlled Trial

**DOI:** 10.3390/jcm15031256

**Published:** 2026-02-04

**Authors:** Merve İşcan Yapar, Neslihan Çelik, Murat Şentürk, Tubanur Çebi Akyüz, Murat Daşhan, Ahmet Kızıltunç

**Affiliations:** 1 Department of Restorative Dentistry, Faculty of Dentistry, Atatürk University, 25240 Erzurum, Türkiye; dtnesli@hotmail.com; 2 Health Services Vocational College, Dental Servıces Department, Iğdır University, 76000 Iğdır, Türkiye; murat.senturk@igdir.edu.tr; 3 Department of Dental Prosthesis Technology, Health Services Vocational School, Atatürk University, 25240 Erzurum, Türkiye; tubanur.cebi@atauni.edu.tr; 4 Department of Biochemistry, School of Medicine, Ataturk University, 25240 Erzurum, Türkiye; drmuratdashan@gmail.com (M.D.); akiziltunc@atauni.edu.tr (A.K.)

**Keywords:** blood pressure, cortisol, dental anxiety, heart rate, music therapy, restorative dentistry

## Abstract

**Background/Objectives**: Dental anxiety is a common clinical problem that negatively affects patient cooperation, treatment acceptance, and physiological stability during dental procedures. This randomized controlled clinical trial study aimed to evaluate the effectiveness of music-based interventions in reducing dental anxiety and stress responses during restorative dental treatment. The null hypothesis was that music exposure would not result in significant differences in anxiety levels or physiological stress parameters compared with standard dental care. **Methods**: Seventy-five patients with moderate to high pre-treatment dental anxiety (MDAS ≥10) were randomly assigned to three groups: classical music, Turkish music, and control (no music) (*n* = 25 per group). Anxiety levels were assessed using the Modified Dental Anxiety Scale (MDAS). Restorations were performed using a standardized adhesive protocol. Physiological parameters, including systolic and diastolic blood pressure (SBP, DBP), heart rate (HR), and oxygen saturation (SpO_2_), as well as salivary cortisol and alpha-amylase levels, were measured before and after restorative treatment. Salivary cortisol and amylase levels were measured using a Human ELISA Kit. Statistical analysis was performed using paired *t*-tests and one-way ANOVA with Tukey’s post hoc test (*p* < 0.05). **Results**: Both music groups showed significant reductions in SBP, DBP, HR, cortisol, amylase, and MDAS scores compared to the control group (*p* < 0.05). Oxygen saturation increased significantly in the music groups, whereas it decreased significantly in the control group. There were no significant differences between classical and Turkish music regarding their anxiety-reducing effects. **Conclusions**: Music-based interventions effectively reduce dental anxiety and physiological stress during restorative dental procedures. This study is novel in simultaneously evaluating subjective anxiety scores and multiple physiological and biochemical stress markers in adult patients undergoing restorative treatment, supporting music as a simple and non-invasive adjunct in clinical dentistry.

## 1. Introduction

Dental anxiety is a complex psychological condition characterized by intense fear and tension related to dental procedures. The reported prevalence of dental fear among adults varies widely in the literature, with estimates ranging from 4% to more than 50% [1]. Dental anxiety has been associated with avoidance or postponement of treatment, poor oral health, and reduced patient and clinician communication. Among dental procedures, restorative treatments are especially anxiety-inducing because of factors like the noise and vibration of rotary tools, fear of pain, and the feeling of losing control during the procedure [2].

Among physiological stress biomarkers, cortisol and alpha-amylase (sAA) levels are commonly used to objectively evaluate autonomic and endocrine responses. Cortisol levels in plasma and saliva rise during stress, making it a biochemical stress hormone [3,4]. Conversely, salivary alpha-amylase indicates sympathetic nervous system activity and serves as a non-invasive marker that quickly increases in response to stress or anxiety [5]. Besides these biochemical indicators, physiological signs such as blood pressure and heart rate also offer important insights into autonomic nervous system activation during stress [6]. Combining these measurements provides a dependable way to monitor both the subjective and objective aspects of anxiety and stress during dental procedures.

Various pharmacological and non-pharmacological strategies have been developed to reduce dental anxiety. Pharmacological approaches include sedative medications, while non-pharmacological interventions consist of relaxation techniques, hypnosis, and various therapy methods such as behavioral therapies, music therapy, and aromatherapy [7,8,9]. Potential side effects and additional costs may limit the use of pharmacological methods, while behavioral interventions require extra training and patient cooperation. As a result, there is increasing interest in non-invasive, cost-effective, and easy-to-implement approaches that do not interfere with routine clinical workflows [10,11].

Music helps by providing psychological relaxation through distraction, masking unpleasant stimuli, and encouraging positive emotional responses, while also affecting physiological processes by regulating the autonomic nervous system and endocrine responses [12,13,14]. These physiological effects can be seen through changes in systolic and diastolic blood pressure (SBP, DBP), heart rate (HR), and oxygen saturation (SpO_2_), as well as decreases in salivary amylase (sAA) and cortisol levels, which are biochemical markers of stress [12,15]. The combined use of subjective and objective measures provides a comprehensive and reliable means to assess how music affects dental anxiety.

Previous studies have shown that listening to music reduces anxiety during pediatric and surgical dental procedures [16,17,18,19]. However, research on the effects of music during restorative treatment on both subjective anxiety scores and physiological parameters is limited. To fill this gap, the present study aimed to evaluate the impact of music-based interventions on psychological (MDAS scores) and physiological (SBP, DBP, HR, SpO_2_, amylase, cortisol) indicators of anxiety during restorative dental treatment. The null hypothesis of this study was that there would be no significant difference in MDAS scores or physiological measurements between patients who listened to music and those who received standard care without music.

## 2. Materials and Methods

### 2.1. Study Design and Ethical Approval

This study was designed as a randomized controlled experimental clinical trial. The research was conducted at the Department of Restorative Dentistry (Atatürk University), between January 2025 and September 2025. The study adhered to the principles of the Declaration of Helsinki on medical protocol and ethics. The study was approved by the Institutional Review Board of Atatürk University Faculty of Medicine Clinical Research Ethics Committee, with the approval date 27 October 2022 and approval no. B.30.2.ATA.0.01.00/480, and the study was supported by the Scientific Research Projects unit of Atatürk University under the application number 12101. Written informed consent was obtained from all participants prior to their inclusion in the study. It was registered in the www.clinicaltrials.gov system under the identifier NCT07173491. This study was reported in accordance with the CONSORT guidelines (see Supplementary Materials, File S1).

### 2.2. Participants and Sample Size

The following inclusion and exclusion criteria were applied to select eligible participants for the study.

#### 2.2.1. Inclusion Criteria

- Systemically healthy adults aged 18–40 years.
- Presence of D2 dentin caries confirmed by clinical and radiographic examination, characterized by radiolucency limited to the outer half of the dentin without pulpal or periapical pathology.
- Indication for single-session restorative treatment.
- Individuals with moderate-to-high dental anxiety according to the Modified Dental Anxiety Scale (MDAS ≥ 10).
- Ability and willingness to complete questionnaires and comply with study procedures.

#### 2.2.2. Exclusion Criteria

- Deep dentin or pulpal involvement lesions, secondary caries, cracks, or fractured teeth.
- Acute or spontaneous dental pain, percussion sensitivity, or periapical pathology.
- Individuals with an MDAS total score ≤ 9 (no/low dental anxiety).
- Hearing impairment or contraindications for music therapy.
- Psychiatric medication use or a diagnosed psychiatric disorder affecting anxiety response.
- Pregnant or breastfeeding individuals.

The required sample size was calculated using G*Power software (Version 3.1.9.4, Heinrich-Heine University, Düsseldorf, Germany), with a test power of 95%, an effect size of 0.5, and a significance level of 5% (α = 0.05). This calculation yielded a minimum required sample size of 45 participants. The effect size was determined based on findings from a previous study evaluating the effectiveness of music distraction interventions in managing dental anxiety [20]. In the present study, to account for potential dropouts and to increase statistical power, a total of 75 participants (*n* = 25 per group) were included.

The primary research question of this study was whether music-based interventions during restorative dental treatment reduce dental anxiety and pain perception compared to standard care.

### 2.3. Intervention Protocol

Following the administration of a questionnaire to gather demographic data (gender, age) and MDAS scores, patients with MDAS scores of 10 or higher (indicating moderate to high dental anxiety) were randomly assigned to three groups (*n* = 25 per group).

In the first group, patients listened to pre-selected classical instrumental music with a slow and relaxing tempo through headphones (JBL T460BT Over-Ear Bluetooth Headphones, Los Angeles, CA, USA) during restorative treatment procedures. In the second group, instrumental Turkish music was played continuously via headphones throughout the restorative procedure. The music volume was adjusted to a comfortable level according to the patient’s preference before the procedure and kept at a level that did not interfere with communication between the clinician and the patient. Patients were able to hear verbal instructions clearly, and communication remained normal throughout the procedure. Patients were also instructed to raise their hand if they needed to communicate or pause the procedure. The music in both groups was played continuously throughout the entire restorative treatment session, from the administration of local anesthesia until completion of the procedure.

The third group served as the control group, where no music intervention was applied during restorative treatments. Routine dental care was provided under standard clinical conditions.

### 2.4. Clinical Procedures

A single carpule of local anesthetic without vasoconstrictor was administered to each patient using a disposable dental syringe. Following caries removal, cavity preparation was performed. The restorative procedure then commenced. All procedures were performed by the same clinician following a standardized protocol. Selective enamel etching was performed with phosphoric acid (Scotchbond, 3M ESPE, Maplewood, MN, USA) for 30 s, followed by rinsing and gentle drying. Universal dental adhesive, Futurabond U single dose (Voco, Cuxhaven, Germany) was applied and polymerized according to the manufacturer’s instructions. Subsequently, the prepared cavities were restored with a resin composite (G-ænial Anterior or Posterior, GC), which was light-cured for 20 s using an LED curing unit (D-Light Duo, GC, Tokyo, Japan). Upon completion of finishing and polishing, the total duration of the restorative procedure was recorded. Only one restoration was performed per patient.

### 2.5. Assessment of Physiological and Salivary Biomarkers

In this study, systolic blood pressure (SBP), diastolic blood pressure (DBP), oxygen saturation (SpO_2_), heart rate (HR), salivary cortisol levels, salivary alpha-amylase (sAA) levels, and MDAS scores were measured initially and after the restorative procedure.

Blood pressure was measured using a manual sphygmomanometer (Perfect, Erka, Bad Tölz, Germany) on the patient’s right arm at heart level, with results given in mmHg. HR and SpO_2_ were checked using a portable pulse oximeter (VZN, Ankara, Turkey) placed on the right index finger.

Unstimulated saliva samples were collected from each participant immediately before and after the procedure. Patients were instructed to expectorate into sterile disposable tubes (Sali-Tubes 100 [SLV-4158], DRG, Marburg, Germany) to ensure the samples were free of blood contamination. To account for circadian fluctuations in cortisol and sAA levels, all samples were collected between 9:00 and 12:00 a.m. The samples were immediately stored at −80 °C until analysis.

A blinded researcher performed all biochemical analyses after the completion of patient treatments. Samples were thawed and centrifuged at 3000× *g* for 10 min. Salivary cortisol levels were measured using a Human Cortisol (COR) ELISA Kit (Catalog No: CK-Bio-11044, Shanghai Coon Koon Biotech Co., Ltd., Shanghai, China). Salivary amylase levels were determined using a Human Amylase (AMS) Kit (Catalog No: CK-Bio-14041, Shanghai Coon Koon Biotech Co., Ltd., Shanghai, China). All analyses were performed according to the manufacturer’s standard protocol using a BIO-TEK ELISA microplate reader (PowerWave XS, BIO-TEK, Winooski, VT, USA) based on the Enzyme-Linked Immunosorbent Assay (ELISA) method. Results were expressed in ng/mL. The intra-assay and inter-assay coefficients of variation (%CV) for the tests were <7% and <10%, respectively.

A total of 50 μL of blanks, standards, and samples was added into the wells pre-coated with amylase/cortisol-specific antibodies. Then, 50 μL of the conjugate solution containing a secondary antibody (except for the blank wells) was added. Following a 1 h incubation at 37 °C, the wells were washed with the washing solution, and 50 μL of avidin-HRP solution was added. After another 60 min incubation at 37 °C, the wells were rewashed, and 50 μL of Chromogen Solution A, followed immediately by 50 μL of Chromogen Solution B, was added to each well. After a 15 min incubation in the dark at 37 °C, 50 μL of Stop Solution was added to each well to terminate the reaction. The absorbance values were then measured at 450 nm. Based on the standard curve generated, the concentrations of amylase and cortisol in the samples were calculated.

### 2.6. Statistical Analysis

Statistical analyses were performed using IBM SPSS Statistics for Windows, Version 27.0 (IBM Corp., Armonk, NY, USA). The Kolmogorov–Smirnov test was applied to assess the normality of the data distribution. Since the data demonstrated a normal distribution, paired-sample *t*-tests were used to compare pre- and post-treatment measurements within each group. For intergroup comparisons, delta values (pre-treatment minus post-treatment values) were calculated, and one-way analysis of variance (ANOVA) followed by Tukey’s post hoc test was performed to identify differences between groups. The statistical significance level was accepted as *p* < 0.05.

Effect sizes (Cohen’s d with 95% CI) were calculated for the pre- and post-measurements. Partial eta squared (ηp^2^) was calculated for one-way ANOVA analyses.

## 3. Results

A total of 75 participants (45 females and 30 males) were included in the study, with 25 participants per group. The gender distribution was comparable across the classical music, Turkish music, and control groups. The mean age was similar among the classical music, Turkish music, and control groups (24–25, 2–24.9). The number of Class II cavities (*n* = 51) restored was greater than that of Class I (*n* = 24) cavities in all groups.

A statistically significant difference was observed in SBP values before and after treatment within and between the groups (Table 1 and Table 2). In both the classical music group and the Turkish music group, SBP significantly decreased following restorative procedures (*p* < 0.001). In contrast, the control group showed a non-significant reduction. When compared between groups, both music interventions produced greater reductions in SBP than the control group (*p* < 0.05), while no significant difference was observed between the two music groups (*p* > 0.05).

Significant within-group changes in DBP were found for both music groups but not for the control group (Table 1). Between-group comparisons revealed that both music-based interventions led to significantly greater reductions in DBP compared with the control group (*p* < 0.05), without a meaningful difference between classical and Turkish music.

Heart rate showed a statistically significant decrease after treatment in both the classical and Turkish music groups (Table 1). No significant change was detected in the control group (*p* = 0.549). Intergroup analysis showed that the decrease in HR was most pronounced in the classical music group, followed by the Turkish music group. The classical music group exhibited a significant difference from both the Turkish music and control groups (*p* < 0.05).

A significant increase in SpO_2_ levels was observed after treatment in classical and Turkish music groups, whereas a statistically nonsignificant decrease was observed in the control group. Between-group comparisons revealed that the changes in SpO_2_ levels at both music groups were significantly different compared with the control group (*p* < 0.05)

The amylase values showed a statistically significant difference before and after the dental treatments in all groups. Between-group analysis confirmed that the decreases were statistically significant compared to the control group (*p* < 0.05), while no statistically significant difference was found between the two music groups (Table 2).

A statistically significant difference was found for cortisol values before and after treatment in all groups (Table 1). Cortisol levels significantly decreased in both the classical and Turkish music groups, while an increase was noted in the control group. Between-group comparisons revealed that post-treatment cortisol levels were significantly lower in both music groups compared to the control group (*p* < 0.05). The Turkish music group showed the most significant reduction in cortisol levels among all groups.

A statistically significant reduction in anxiety scores, as measured by the MDAS, was observed between pre- and post-treatment assessments in all groups (Table 1). When compared across groups, the greatest MDAS scores reduction occurred in the classical music group, followed by the Turkish music group, both of which differed significantly from the control group (*p* < 0.001) (Table 2).

## 4. Discussion

Dental anxiety is a multifactorial condition that adversely affects oral health by causing treatment avoidance, poor cooperation, and reduced clinical success, and is associated with increased physiological stress responses during dental procedures [12,21,22]. Therefore, there is increasing interest in non-pharmacological interventions, such as music therapy, as safe, cost-effective, and easily applicable approaches to reduce dental anxiety [23,24,25]. The present study aimed to evaluate the effectiveness of music-based interventions in lowering dental anxiety and physiological stress responses during restorative dental procedures. The findings showed statistically significant improvements in both physiological and psychological outcomes among participants who listened to music, compared to the control group. Therefore, the null hypothesis was rejected.

In recent years, growing evidence has continued to support the effectiveness of music-based interventions in reducing dental anxiety and physiological stress responses, particularly in adult patient populations. Recent studies have reported significant reductions in anxiety scores, heart rate, and blood pressure among patients exposed to music during restorative and surgical dental procedures, emphasizing music as a simple yet effective adjunct to routine dental care [2,26]. These contemporary findings reinforce earlier observations while highlighting an increasing clinical interest in non-pharmacological anxiety management strategies that are easy to implement and well accepted by patients. The results of the present study are consistent with this recent body of literature, further confirming the anxiolytic and stress-modulating effects of music during restorative dental treatment.

Furthermore, recent investigations have emphasized the importance of combining subjective anxiety assessments with objective physiological and biochemical markers to achieve a more comprehensive understanding of stress regulation during dental procedures. Studies conducted within the last few years have demonstrated that music exposure not only reduces self-reported anxiety but also significantly modulates salivary cortisol, alpha-amylase levels, and cardiovascular parameters, suggesting a multidimensional effect on both psychological perception and autonomic nervous system activity [25,27]. By simultaneously evaluating MDAS scores alongside cardiovascular, respiratory, and salivary biomarkers, the present study aligns with these contemporary methodological approaches and contributes updated evidence supporting the integration of music-based interventions into restorative dental practice.

Regarding cardiovascular responses, both classical and Turkish music led to significant reductions in SBP and DBP compared with baseline values and the control group. This finding aligns with studies suggesting that slow-tempo, rhythmically stable music promotes parasympathetic activation and decreases sympathetic arousal, thereby lowering blood pressure and heart rate [28,29]. Mejía-Rubalcava et al.’s [12] study results showed a significant decrease in blood pressure in the music therapy group. They attributed this decrease to the influence of music therapy on the Autonomic Nervous System, which could reduce the outlying vascular resistance by decreasing sympathetic stimulation of the blood vessels. Di Nasso et al. [30] report that music therapy reduced systolic and diastolic blood pressure and heart rate during root canal treatments. In our study, HR decreased markedly in both music groups, with classical music producing a slightly greater reduction. This outcome supports earlier research demonstrating that classical compositions characterized by moderate tempo and predictable melodic patterns effectively stabilize cardiac rhythm and promote physiological calmness [23,31]. Pellicer et al. [32] noted that heart rate (HR) increases due to endogenous epinephrine released from the adrenal medulla in response to anxiety or pain during dental treatments, and their study showed that classical music lowers heart rate more than other types of music.

SpO_2_ levels rose significantly after music exposure, especially in the Turkish music group, indicating improved respiratory efficiency and relaxation. This increase may be due to reduced muscle tension and slower breathing patterns caused by listening to calm, culturally familiar melodies [33]. A study carried out on pediatric individuals with chronic diseases observed a significant increase in oxygen saturation of 2–3% on average following music therapy, indicating that music has a positive effect on respiratory efficiency and relaxation [34]. Similar improvements in SpO_2_ following music interventions have been observed in medical and dental settings, showing reduced sympathetic dominance and better oxygen exchange efficiency [35,36].

Salivary biomarkers provided additional evidence of stress modulation through music. Salivary amylase, a sensitive marker of sympathetic nervous system activity, significantly decreased after treatment in both music groups, while it slightly increased in the control group. These results align with studies indicating that music reduces catecholamine release and lowers salivary amylase levels, reflecting decreased stress-related sympathetic arousal [37,38]. Ooishi et al. [39] reported a significant reduction in salivary cortisol levels following exposure to relaxing music, indicating a suppression of hypothalamic–pituitary–adrenal axis activity. Similarly, Karapicak et al. [2] observed decreased cortisol concentrations in patients with moderate dental anxiety during restorative procedures when music was played. In the present study, a similar trend was observed, supporting the idea that music can reduce physiological stress by influencing endocrine responses. Overall, these findings bolster previous evidence that music has measurable effects on both sympathetic nervous system activity and stress markers associated with the HPA axis.

Psychological assessment using the Modified Dental Anxiety Scale (MDAS) showed significant reductions in anxiety scores for both music groups compared to the control group. Classical music resulted in the most notable improvement, supporting previous evidence that structured harmonic compositions can influence cognitive and emotional processes related to anxiety [33]. In a study evaluating the effect of music on dental anxiety, a significant decrease in MDAS scores was observed in the music therapy group, suggesting that music may be effective in reducing anxiety [40]. Another randomized controlled study of adults undergoing restorative dental treatment found that listening to music was associated with a significant reduction in Modified Dental Anxiety Scale scores compared to the control group [2]. Overall, the decrease in both subjective (MDAS) and objective (physiological) stress indicators confirms that music has a positive effect during dental procedures.

Several limitations of this study should be acknowledged. First, only two types of music were evaluated. Different musical genres may exert varying psychological and physiological effects; therefore, the findings may not be generalizable to other music-based interventions. In addition, other non-pharmacological anxiety-management strategies were not included, which limits comparisons with alternative approaches. Although some studies have reported enhanced anxiety reduction when participants select their own music, individual music preferences were not assessed in the present study. Another limitation is the absence of a placebo auditory control condition, such as neutral sounds or white noise. Consequently, expectancy effects associated with wearing headphones cannot be entirely ruled out. Furthermore, participants were aware of whether they received a music intervention, as blinding was not possible due to the nature of the study design. This may have introduced performance and placebo bias. Future studies incorporating placebo-controlled or blinded auditory conditions may provide a more rigorous evaluation of the specific effects of music on dental anxiety and help to minimize these potential biases.

## 5. Conclusions

Within the limitations of this study, music-based interventions significantly decreased dental anxiety and stress responses among patients undergoing restorative dental treatment. Both classical and Turkish music were associated with improvements in cardiovascular parameters, salivary stress biomarkers, and MDAS anxiety scores. These findings support that music can serve as a non-invasive, cost-effective, and efficient adjunct tool to enhance patient comfort and physiological stability during dental procedures. Future studies should investigate the long-term effects of music-based interventions, compare different musical genres, and consider individual music preferences to optimize personalized anxiety management in dental settings.

## Figures and Tables

**Table 1:** Within-Group Comparisons of Pre- and Post-Treatment Outcome Measures.

	Classical Music				Turkish Music				Control			
	Before Mean ± Std Deviation	After Mean ± Std Deviation	*p* Value *	Cohen’s d	Before Mean ± Std Deviation	After Mean ± Std Deviation	*p* Value *	Cohen’s d	Before Mean ± Std Deviation	After Mean ± Std Deviation	*p* Value *	Cohen’s d
SBP (mmHg)	108.8 ± 13.4	100.2 ± 11.6	**<0.001**	1.52	116.4 ± 10.1	106.2 ± 11.5	**<0.001**	1.67	115.1 ± 14	111.5 ± 10.3	0.06	0.40
DBP (mmHg)	75.3 ± 6.9	71.6 ± 6	**<0.001**	0.99	80.1 ± 9.0	75.5 ± 8.1	**0.002**	0.69	81.0 ± 8.7	81.3 ± 8.7	0.855	0.04
HR (beats per minute-bpm)	94 ± 10.9	82.7 ± 9.5	**<0.001**	2.01	96.2 ± 4.9	91.6 ± 3.0	**<0.001**	1.24	86.2 ± 15.1	85.0 ± 13.0	0.549	0.12
SpO_2_ (%)	92.4 ± 4.9	96.6 ± 3.8	**<0.001**	1.28	95.7 ± 1.9	96.7 ± 1.4	**0.016**	0.52	94.7 ± 2.9	93.2 ± 4.0	0.067	0.38
Amylase (ng/mL)	22.4 ± 3.6	18.8 ± 3.3	**<0.001**	1.17	22.7 ± 5.7	18.3 ± 4.9	**<0.001**	1.03	23.4 ± 3.5	25.1 ± 3.0	**0.019**	0.51
Cortisol (ng/mL)	25.7 ± 8.9	20.8 ± 8.6	**<0.001**	1.40	31.7 ± 15.1	24.6 ± 9.8	**<0.001**	0.91	24.7 ± 9.1	27.8 ± 8.1	**0.011**	0.55
MDAS (scale score)	17.5 ± 2.8	9.9 ± 2.7	**<0.001**	2.12	16.1 ± 2.6	11.9 ± 3.6	**<0.001**	1.51	14.9 ± 1.9	14.4 ± 1.6	**0.008**	0.58

* paired-sample *t*-tests statistics; the bold *p*-values indicate statistical significance.

**Table 2:** Intra-Group Comparisons of Δ values Outcome Measures.

	Classical Music	Turkish Music	Control	Partial n^2^
ΔSBP	8.6 ± 5.7 ^A^	10.2 ± 6.1 ^A^	3.6 ± 9.1 ^B^	0.139
ΔDBP	3.7 ± 3.7 ^A^	4.6 ± 6.6 ^A^	−0.3 ± 7.5 ^B^	0.108
ΔHR	11.3 ± 5.6 ^A^	4.6 ± 3.7 ^B^	1.2 ± 9.9 ^B^	0.278
ΔSpO_2_	−4.2 ± 3.2 ^A^	−1.0 ± 2.0 ^B^	1.5 ± 3.9 ^C^	0.359
ΔAmylase	3.6 ± 3.1 ^A^	4.4 ± 4.3 ^A^	−1.7 ± 3.3 ^B^	0.370
ΔCortisol	4.9 ± 3.5 ^A^	7.1 ± 7.7 ^A^	−3.1 ± 5.6 ^B^	0.365
ΔMDAS	7.56 ± 3.6 ^A^	4.2 ± 1.8 ^B^	0.5 ± 0.8 ^C^	0.609

The Δ values represent the difference between pre- and post-treatment measurements. The capital letters indicate statistically significant differences between the groups. (ANOVA) followed by Tukey’s post hoc test).

## Data Availability

Data is available from the authors upon reasonable request.

## Supplementary Materials

The following supporting information can be downloaded at: https://www.mdpi.com/article/10.3390/jcm15031256/s1. File S1: CONSORT checklist. Reference [41] is cited in the Supplementary Materials.

## Abbreviations

The following abbreviations are used in this manuscript:

MDAS	Modified Dental Anxiety Scale
SBP	Systolic blood pressure
DBP	Diastolic blood pressure
HR	Heart rate
SpO_2_	Oxygen saturation
sAA	Salivary amylase
μL	Microliter
nm	Nanometer
ng/mL	nanograms per milliliter
mmHg	millimeters of mercury
